# Crystal engineering in IUCrJ: from ‘the’ crystal structure to ‘a’ crystal structure

**DOI:** 10.1107/S2052252521000993

**Published:** 2021-02-20

**Authors:** Gautam R. Desiraju

**Affiliations:** a Solid State and Structural Chemistry Unit, Indian Institute of Science, Bangalore, 560 012, India

**Keywords:** crystal engineering, crystal structure, crystal landscapes, editorial

## Abstract

What is ‘structure’ in the context of a molecular solid?

Crystal engineering continues to grow at an astonishing pace and one even discerns the appearance of offshoots that are beginning to develop as subjects in their own right, in the same way that crystal engineering itself developed as an offshoot of supramolecular chemistry in the 1990s. **IUCrJ** has successfully captured some of these latest developments in the 30 odd papers published in the journal in 2020 and early 2021. These papers encapsulate the idea of ‘structure’ as applied to molecular organic and metal–organic crystals. What is ‘structure’? How does ‘structure’ relate to property? As one becomes more adept in designing structures, does one develop a parallel felicity in designing properties?

Until the end of the 1980s, the focus in small molecule crystallography was the determination of the unknown crystal structure of a molecular solid, which was typically obtained as a stable, well formed crystal that diffracted well and wherein the solution and refinement of the structure, in other words the (*x*, *y*, *z*) of all the constituent atoms and their atomic displacement parameters (ADPs), constituted the entirety of the exercise. The story, as it were, ended with obtaining these (*x*, *y*, *z*) and ADP values and given the emphasis then on the molecular structure of an organic compound, the exercise was deemed to be over when crystallography confirmed or, in some cases, corrected the molecular structure as obtained by chemical and spectroscopic methods. This was ‘the’ crystal structure (Clegg, 2021 ▸).

Starting with the 1990s, a new story started, one that began rather than ended with the (*x*, *y*, *z*) of all the atoms in the unit cell, and thus began the modern version of the subject of crystal engineering, a term that was coined in the mid-1950s but used in a limited and restricted sense until the 1990s. Why does a molecule crystallize the way it does? Why does a molecule not crystallize in some other way? Why do some molecules form good crystals and others not? Why do the habits of molecular crystals show so much variability? And most important, why do some compounds crystallize with more than one crystal structure – polymorphism?

Polymorphism (Broadhurst *et al.*, 2020 ▸; Shi *et al.*, 2020 ▸) was the beginning of a new theme that conceives of a structure determined by crystallography as just that: a structure. Any given pure chemical compound can have more than one stable crystal structure. Gradually, this idea of a one-to-many correspondence between molecular and crystal structure extended to a compound having metastable and transient crystal forms as well, and finally *in silico* forms that were still to be isolated experimentally. This breakdown of the one-to-one correspondence between molecule and crystal also occurred in the reverse direction. Different molecules could have what is essentially the same crystal structure, isomorphism (Ranjan *et al.*, 2020 ▸), and so an *in silico* structure of a given compound, which might not have been realized experimentally, might well be seen experimentally in a closely similar analog. For example, chloro groups could be replaced by methyl or bromo groups, or even by a nitro group; an ethene linkage could be replaced by a sulfur atom; an (*R*)-*sec*-butyl group could be replaced by an (*S*)-*sec*-butyl group, and so on. In the end, things that were known earlier, were rediscovered in a new context, a not uncommon occurrence in science. And so, by using the device of solid solution formation (Verma *et al.*, 2020 ▸), one could engineer the crystal structure of a given compound into a computer-generated structure through solid solution formation with a congener that displays the desired structure experimentally in its pure state. Effectively, the idea could be extended: a two-component crystal could have the crystal structure of a single-component crystal. All these ‘structures’ of a single organic compound effectively constitute the structural landscape. Any structure determined in the laboratory with crystallography is a data point in this landscape. A compound is associated not with one unique crystal structure but with many. Any of these is ‘a’ crystal structure in this landscape. And thus, crystal engineering facilitated this conceptual shift in thinking from *the crystal structure* to *a crystal structure*.

One of the papers published in **IUCrJ** in 2020 puts this in a colourful way. Talking about olanzapine, Reutzel-Edens & Bhardwaj (2020 ▸) refer to their report as ‘painting what is a partial picture of the amazingly complex crystal chemistry of this important drug molecule’. Yes, a landscape needs to be painted and what one needs in the palette is a combination of high throughput crystallization and high throughput crystallography. What we need are databases not of crystal structures but of crystal landscapes. Crystal structure prediction (CSP) should graduate into developing a library of landscapes. *In silico* structures and virtual supramolecular synthons are already a reality in the armoury of the crystal engineer and so the subject advances.

**IUCrJ** published 30 papers in the Crystal Engineering and Chemistry section in 2020 out of the total of 165 in this section since the inception of the journal in 2014. **IUCrJ** published 130 papers in all sections last year as compared to the 650 that the journal has published in its entirety since its inception in 2014. So, the chemistry component in 2020 is just about its running average across all years, but it must be noted that we have more sections now than we did in 2014. The papers in our section continue to contribute their share to reaching an impact factor of 5.401 in 2019, which is an impressive increase over the previous year.

In keeping with the theme of ‘structure’ a number of papers dealt with cocrystals both in the pharmaceutical context (Fatima *et al.*, 2020 ▸; Ranjan *et al.*, 2020 ▸) and in a more general sense (Rajkumar & Desiraju, 2021 ▸). Other areas addressed included charge density (Chodkiewicz *et al.*, 2020 ▸; Sanjuan-Szklarz *et al.*, 2020 ▸; Duverger-Nédellec *et al.*, 2020 ▸), phase transformations (Smets *et al.*, 2020 ▸; Rekis *et al.*, 2021 ▸), crystals obtained under exotic conditions (Buganski & Bindi, 2021 ▸; Hu *et al.*, 2020 ▸; Németh, 2020 ▸), and MOFs (Yadava *et al.*, 2020 ▸; Park *et al.*, 2020 ▸) apart from core papers on supramolecular synthons and synthetic strategy.

I would like to see more papers in this section in 2021–2022 as the journal gradually moves into the latter part of its first decade. Publishing in **IUCrJ** is prestigious even as the relevance of open access continues to strengthen and grow. The Co-editors in this section have contributed significantly especially with the trials and tribulations of the pandemic that we seem to be getting through. Publication times have not been seriously affected and I owe all of them a big thank you.
